# Editorial: Dissecting Traditional Medicine *via* Chemical and Biochemical Techniques: Advanced Analytics and Novel Paradigms

**DOI:** 10.3389/fphar.2022.839004

**Published:** 2022-02-08

**Authors:** Yi Wang, Peng Li, Mirko Baruscotti

**Affiliations:** ^1^ College of Pharmaceutical Sciences, Zhejiang University, Hangzhou, China; ^2^ State Key Laboratory of Quality Research in Chinese Medicine, Institute of Chinese Medical Sciences, University of Macau, Taipa, China; ^3^ Department of Biosciences, University of Milano, Milan, Italy

**Keywords:** Chinese medicine, LC-MS, ethnopharmacology, system biology, traditional Chinese medicine

Traditional medicine (TM) plays an important role in the healthcare system and is increasingly used worldwide for managing various chronic diseases and promoting well-being. Although huge progresses have been achieved to reveal major compounds in well-known botanical drugs, such as *Salvia miltiorrhiza* Bunge, the identification of major active constituents remains extremely challenging due to the complexity of chemical composition and the elusive mechanisms of action. One major barrier to solving such problems is the lack of feasible techniques and strategies to distinguish those active compounds from those which make no or only a minor contribution to the activity of TM.

Often ubiquitous or widely distributed compounds are then claimed to be “actives” for major disease conditions. Moreover, it is difficult to judge whether certain components or compounds identified by a specific cellular model or target-based assays can exert a pharmacological effect *in vivo* in the context of biological network or in a real-life clinical setting. Therefore, it is time to dedicate greater efforts in either improving the capability of bioassays or developing novel paradigms to refresh our toolbox for dissecting TMs. The aim of this research topic is to improve the quality of such studies, accelerate the pace of screening and identification of active compounds from TMs, and to facilitate the expansion of screening assays from cellular tissue levels to organs and *in vivo* models.

In the past 3 decades, bioactivity-guided screening has become a mainstream workflow for lead discovery from TMs. With the aid of modern analytical approaches such as LC-MS, GC-MS, and NMR, it allows us to identify multiple constituents in active components found by phenotypes or target-based assays. However, synergistic effects among multiple compounds of TMs are difficult to recognize during conventional phytochemical isolation, which requires repeated purification steps to obtain a single compound from mixtures of TM. In this research topic, four articles are dedicated to develop novel analytical or preparing approaches to uncover the chemical composition of TM. Nie et al. used the emerging microscopic mass spectrometry imaging technique to reveal the distribution of phytochemicals in the dried root of *Isatis tinctoria* L. With the aid of the chemometric method, different samples from four habitats were successfully clustered. Chang YX and co-workers Yang et al. established a rapid high throughput vibration and vortex-assisted matrix solid phase dispersion method for simultaneous extraction of four isoflavones from *Glycine* max (L.) Merr. With satisfied recovery and a linear range, the approach was applied in the quality control of SSP.

As the mainstream technique for analyzing complex extracts of TM, LC-MS was also widely used in metabolite profiling. Zhang et al. analyzed the major compounds of the Buxue Yimu granule (BYG) and the serum endogenous metabolites regulated by the BYG. By integrating the metabolomic and biochemical data, they concluded that the BYG exerts a potential protective role in the intervention of incomplete abortion by anti-inflammation, promotes endometrial repair, and regulates the metabolic disorders. The study by Houriet et al. presented a comprehensive MS-based analytical workflow that allowed a generic and unbiased selection of specific and abundant markers and the identification of multiple related sub-markers, which is critical for metabolite profiling of TM. In a clinical study by Li et al., the serum metabolic profiles of 45 healthy controls and 124 patients with psoriasis vulgaris (PV) were analyzed. A total of 14 biomarkers related to syndrome differentiation and psoriasis types were identified, which might be potential biomarkers for diagnosis. Except for the metabolomics study, the gut microbiome is another hot topic in pharmacology of TM Cui et al. investigated the protective effects of Da-Chai-Hu decoction (DCH) against nonalcoholic fatty liver disease (NAFLD). 16S rRNA sequencing and untargeted metabolomics suggested that DCH regulated the arachidonic acid (AA), glycine/serine/threonine, and glycerophospholipid metabolic pathways and altered the abundance of several key gut microflora.

Multiple novel pharmacological techniques, e.g., high-throughput screening and high-content screening, provide opportunities to compare the efficacy of components singularly and as a whole on a larger scale. The screening models are shifting from the conventional biochemical and cellular assay to co-culture models, 3D-cultured organoid, organ-on-a-chip, and *in vivo* zebrafish model (small animal models).

Li et al. identified two representative compounds i.e., cryptotanshinone and senkyunolide I from the botanical drug Guanxinning Table. Their findings showed that the synergistic anti-thrombotic effect of two compounds from different herbs can be attributed to the regulation of thrombi formation at different levels *via* multiple signaling pathways, including oxidative stress, platelet activation, and coagulation cascade. Ma et al. also utilized *in vivo* zebrafish to evaluate anti-thrombosis effects of Xuesaitong injection (XST); another widely used botanical drug consisted of several saponins from *Panax notoginseng* (Burkill) F.H.Chen. They found that XST downregulates the expression of the fibrinogen alpha chain (fga) gene to inhibit the coagulation cascade during thrombosis, while it can be applied to evaluate the batch-to-batch consistency of XST. The study by Zhang et al. established a xenograft zebrafish model to investigate the anticancer effects of theaflavin (TF), a major active pigment of tea. Combined with *in vitro* effects, they demonstrated that TF possessed cytotoxic pro-apoptotic and tumor-inhibitory effects on A375 cells through activations of P53 and JNK pathways.

This issue also contains several articles focused on active substances of TM for various diseases. Berberine is a well-known benzylisoquinoline alkaloid in *Coptis chinensis* Franch. The study by Zhai et al. demonstrated for the first time that berberine directly binds to PLA2G4A and inhibits the MAPK/JNK signaling pathway to inhibit the PLA2G4A activity in inflammatory status. Chen et al. investigated the protective effect of pure total flavonoids from citrus (PTFC) against non-steroidal anti-inflammatory drug (NSAID)-induced intestinal injury *in vivo* and *in vitro*. They found PTFC promoted autophagy through the PI3K/Akt signaling pathway to protect the intestinal barrier integrity. Another study by Liu et al. evaluated the anti-hepatoma compound in *Heptapleurum heptaphyllum* (L.) Y.F.Deng by the component knock-out strategy. The compound A exerts the anticancer effect by regulating the levels of ROS and Bcl-2 family protein in the mitochondrial apoptosis pathway. Lu et al. identified a class of unique cyclic-peptide from *Pseudostellaria heterophylla* (Miq.) Pax, which is a commonly used TM for treating lung diseases. It is the first report of those cyclic peptides that improve the pulmonary ventilation function in the rat model of chronic obstructive pulmonary disease (COPD). The mechanism of action is attributed to inhibiting the abnormal activation of the TLR4-MyD88-JNK/p38 pathway.

In summary, the combined use of abovementioned novel methods and strategies will prompt the efficacy of modern research of TM. We hope that readers will find inspiration from this research topic. Therefore, the discovery of active compounds in TM will move further away from the systematic isolation and bioassay-guided isolation to programmed/targeted isolation to find those parts with equivalent effects as a whole, improving our pharmacological understanding of complex mixtures.

